# Radio Electric Asymmetric Conveyer: A Novel Neuromodulation Technology in Alzheimer’s and Other Neurodegenerative Diseases

**DOI:** 10.3389/fpsyt.2015.00022

**Published:** 2015-02-17

**Authors:** Salvatore Rinaldi, Laura Calzà, Luciana Giardino, Gabriele E. M. Biella, Antonio G. Zippo, Vania Fontani

**Affiliations:** ^1^Rinaldi Fontani Foundation, Florence, Italy; ^2^Department of Neuro Psycho Physical Optimization, Rinaldi Fontani Institute, Florence, Italy; ^3^Department of Regenerative Medicine, Rinaldi Fontani Institute, Florence, Italy; ^4^IRET Foundation, Ozzano dell’Emilia, Italy; ^5^Interdepartmental Center for Industrial Research (HST-ICIR), University of Bologna, Bologna, Italy; ^6^Institute of Bioimaging and Molecular Physiology, National Research Council, Segrate, Italy

**Keywords:** Alzheimer’s disease, brain stimulation, brain modulation, neurodegenerative disease, senescence, motor disorders, regenerative medicine, REAC technology

## Abstract

Global research in the field of pharmacology has not yet found effective drugs to treat Alzheimer’s disease (AD). Thus, alternative therapeutic strategies are under investigation, such as neurostimulation by physical means. Radio electric asymmetric conveyer (REAC) is one of these technologies and has, until now, been used in clinical studies on several psychiatric and neurological disorders with encouraging results in the absence of side effects. Moreover, studies at the cellular level have shown that REAC technology, with the appropriate protocols, is able to induce neuronal differentiation both in murine embryonic cells and in human adult differentiated cells. Other studies have shown that REAC technology is able to positively influence senescence processes. Studies conducted on AD patients and in transgenic mouse models have shown promising results, suggesting REAC could be a useful therapy for certain components of AD.

## Introduction

Global research in the field of pharmacology has not yet found effective drugs to treat Alzheimer’s disease (AD). As an alternative, new therapeutic strategies are under investigation and clinical testing. In particular, neurostimulation by physical means is regarded as a promising strategy for symptomatic therapies for memory and psychiatric symptoms (1), and also to counteract amyloid accumulation (2) and modulate neuroplasticity (3).

Currently, the neurostimulation technologies most prevalent in psychiatry are transcranial magnetic stimulation (TMS), deep brain stimulation (DBS), vagus nerve stimulation (VNS), and transcranial direct current stimulation (tDCS). A new technology for bio and neuromodulation has been recently developed, termed radio electric asymmetric conveyer (REAC) technology (4, 5).

Until now, REAC technology has been used in clinical studies on several psychiatric and neurological disorders with encouraging results in the absence of side effects (6–15). Studies have also been conducted in AD patients with promising results (16–18). Moreover, studies at the cellular level have shown that REAC technology, with the appropriate protocols, is able to induce neuronal differentiation both in murine embryonic cells and in human adult differentiated cells (19–21). Other studies have shown that REAC technology is able to positively influence senescence processes (22, 23).

## Similarities and Differences between REAC Technology and Other Neurostimulation–Neuromodulation Techniques

REAC technology is based on the use of radio fields concentrated in an asymmetrical way. This innovative technology (1, 2) uses very weak emissions in the radio frequency range, usually 2.4, 5.8, or 10.5 GHz. The current models of REAC devices use only 2.4 or 5.8 GHz emissions, such as those issued by common Wi-Fi devices. These particular characteristics of REAC technology make it completely distinct from all other neurostimulation–neuromodulation technologies (24).

### REAC technology operating characteristics

REAC technology is independent of the radio frequency emission used. REAC devices use only frequencies of very weak intensity (2.4 and 5.8 GHz) because these are the most widely used and permitted at the international level (i.e., Wi-Fi). REAC technology is effective at very low power levels – approximately 2 mW at the emitter. Higher powers alter its mechanism of action. Emission power is typically kept constant for all protocols, and instead, the number of pulses and the duty cycle are varied.

The peculiarity of REAC technology is not the emission, but the particular physical link between the device and the patient’s body. This link is represented by the probe-asymmetric conveyer. This is the ultimate innovation of REAC technology, covered by international patents (4, 5) and other patents in progress. Solely thanks to the conveyer (asymmetric probe), the REAC emission can interact with the biological tissues of the human body without depth limits and in a very innovative way. This interaction generates induced radio frequency microcurrents in tissues that vary according to their molecular characteristics. The sum of these induced radio frequency microcurrents gives rise to a resulting microcurrent generated by the tissue of the subject. This resulting microcurrent, which we can term “autologous,” is concentrated by the probe-asymmetric conveyer of the device and exerts a therapeutic effect, which opens up new perspectives in the ambit of treatments through physical devices.

Thanks to the peculiarities of its mechanism of action, REAC technology has no limits of action related to the depth of the tissue to be treated, and thus, organs deep within the body can also be treated. Moreover, due to the nature of its mechanism of action, REAC technology is safe, painless, and non-invasive. Its application is rapid and has no contraindications or known side effects.

#### Administration methods

REAC technology’s key feature is to concentrate the induced microcurrents that are generated in the body. Therapeutic treatments with REAC technology are administered by the application of the cable probe of the REAC device on specific points of the body. The duration of each treatment depends on the type of protocol used and varies from the millisecond to hour timescale.

Until now, REAC therapies have utilized two neuromodulation protocols, Neuro Postural Optimization (NPO) and Neuro Psycho Physical Optimization (NPPO). The NPO protocol consists of a single 250 ms REAC pulse and is designed to determine initial efficacy of neuromodulation. This effect is particularly stable over time and capable of determining positive neuromotor effects, subjective and objective, detectable with functional magnetic resonance imaging (fMRI) (25–27). The NPPO consists of seven 500 ms REAC pulses, administered in a precise sequence. Each NPPO treatment cycle consists of 18 sessions. This NPPO protocol has been shown to result in the improvement of various mental and psychiatric disorders (7–10, 12–15).

Currently, it is not possible to determine how many treatment cycles are necessary to prevent or counteract the progression of AD; however, we assume that the therapeutic approach should be chronic, specifically three to four NPPO treatment cycles per year.

New therapeutic treatments for regenerative medicine are currently being tested in animal models.

## REAC Technology Efficacy

### Long-lasting changes in brain activation induced by REAC technology pulses and efficacy in motor control

In fMRI studies (25–27) on healthy subjects, brain activity has been shown to be sensitive to REAC neuromodulation treatment administered according to the NPO. REAC-NPO is thought to modulate and optimize motor and postural strategies (28), thus inducing neuromodulation. It is possible to highlight this neuromodulation though the fMRI BOLD signal via reductions in signals from motor regions activated by simple motor tasks like finger tapping (26) or leg flexion (25, 27). These BOLD signal spatial reductions in REAC-NPO-treated groups are particularly noticeable in cerebellar and ponto-mesencephalic areas, where the signal completely disappears.

These findings, which can be interpreted as a functional optimization of the brain structures that govern the coordination of motor control and balance, led us to hypothesize that REAC-NPO could be useful in the treatment of motor disorders. In AD patients, motor disorders are more evident in the later stages of the disease, when deficits in gait and balance, as well as other manifestations of motor deterioration, emerge. This condition has direct consequences on quality of life and patient and caregiver suffering. On this basis of these observations, we, therefore, investigated whether the positive results of REAC-NPO treatment could also be obtained in patients with advanced AD (17).

We obtained confirmation of our hypothesis in randomized, controlled trials where we also investigated the feasibility, safety, and short-term motor effects of brain stimulation with REAC-NPO in patients with advanced AD. The results showed that REAC-NPO improved gait and number of steps per second. These results hold some promise for a role of REAC in the treatment of motor deterioration in AD. In fact, on the basis of its non-invasive, painless, and safe nature, REAC seems particularly attractive for treating patients with advanced AD who are often elderly and frail.

### REAC electrophysiological effects on neural activity in the somatosensory thalamocortical loop

The effects of REAC neuromodulatory protocols on neuronal spiking and synaptic (local field potential) activity have been recorded simultaneously from two brain regions – the somatosensory thalamus and cortex. The application of REAC in rats during electrophysiological recordings showed significant effects both on normal animals and an experimental model of chronic pain (the chronic constriction injury, or CCI, model) with anomalous neuronal discharge patterns – namely increases in discharge rate, hyper-responsiveness to light and noxious stimuli, and long after-discharges after stimulus withdrawal, all complements to the semeiotic clinical signs of allodynia, hyperalgesia, and persistent pain after stimulus discontinuation.

The effects of REAC, evident both in spontaneous and stimulus-evoked activity, were characterized by significant increases in spiking frequency (>25–30% of the overall mean firing rate) and local field potential synchrony in both control and CCI animals. Overall, these results suggest profound influences of REAC on brain functional network architecture at the cellular level, even in strongly perturbed networks such as the CCI chronic pain model. These findings at the neuronal scale supplement the previous fMRI data that reported REAC-induced BOLD signal reductions in motor regions activated by simple motor tasks involving finger tapping (26), leg flexion (27), and postural balance control (28).

### REAC cognitive effects on AD

The NPPO protocol has demonstrated significant efficacy in improving psychiatric disorders such as anxiety, depression, and bipolar disorder. Based on these results, we hypothesized that NPPO could be helpful in the treatment of behavioral symptoms and cognitive functioning in AD patients and in several forms of behavioral and cognitive-impairment disorders.

In a study conducted on eight patients diagnosed with AD according to the DSM-IV-TR criteria, and presenting with behavioral and/or psychiatric disturbances, we observed that the REAC-NPPO protocol is able to enhance cognitive and behavioral functioning. All measures of cognitive functioning were significantly improved after the initial REAC-NPPO treatment and continued to improve after the subsequent REAC-NPPO treatment cycle. Similarly, all behavioral measurements were positively affected after the first REAC-NPPO treatment, and most continued to improve after the subsequent REAC-NPPO treatment cycle. We also confirmed that REAC-NPPO treatment has a high safety and tolerability profile. Based on these observations, we hypothesize that REAC-NPPO treatment may be useful not only for AD patients but also for other forms of dementia (i.e., vascular dementia or mixed conditions). Further studies are needed to confirm this hypothesis.

### Studies in animal models of AD

REAC technology is also under study in animal models of Alzheimer disease. In particular, the effects of REAC exposure on the main landmark symptoms of AD (i.e., learning and memory impairment, as well as amyloid deposition and neuroinflammation) are being investigated in Tg2576 mice. Tg2576 mice overexpress the Swedish double mutant form of APPswe695 (K670N-M671L) under the hamster PrP promoter, on a background of C57BL/6 and SJL mouse strains (29) and is a widely used model to test novel AD treatments in a translational approach (30, 31). Learning and memory deficits appear quite early in this mouse model, well before plaque deposition (32), and are associated with synaptic and neurotransmitter dysfunctions involving acetylcholine, GABA, and glutamate (33, 34). In old and very old animals, classical amyloid plaque deposition, tau hyper-phosphorylation, and neuroinflammation (34) are evident. In these very old animals, we have tested the effects of 2-week exposures to REAC (6 h per day) on learning and memory, locomotion, amyloid deposition, and neuroinflammation. Preliminary results indicate that REAC exposure increases spontaneous locomotion and dramatically decreases neuroinflammation, as detected by decreased expression of the microglia marker Iba1 and the astroglial marker GFAP. These preliminary results suggest that REAC is an effective technology for decreasing neuroinflammation associated with amyloid plaque deposition.

### REAC induces direct neuronal differentiation in murine embryonic and human differentiated cells

The clinical results obtained with REAC technology led us to investigate the biological and molecular mechanisms that would allow us to understand the clinical outcomes of its use. Studies were conducted with specific protocols for cell culture in order to investigate REAC efficacy at the cellular level. In particular, we investigated the effects of REAC stimulation on the transcription of neurogenin1 during neurogenesis in murine embryonic (ES) (21) and human differentiated cells (19, 20). We provided evidence that REAC exposure resulted in the induction of targeted genes controlling neurogenic and myogenic commitment, followed by the appearance of the corresponding phenotypes, in mouse ES cells. Moreover, we demonstrated the effectiveness of REAC treatment in downregulating expression of the genes controlling pluripotency in mouse ES cells after 24 h of exposure.

We exposed cells to REAC by immersing the conveyer electrodes in the culture medium. Cell responses were investigated by real-time PCR, Western blot, and confocal microscopy. RF burst duration, radiated power, electric and magnetic fields, specific absorption rate, and current density in culture medium were monitored. REAC stimulation primed transcription of genes involved in cardiac (GATA4, Nkx-2.5, and prodynorphin), skeletal muscle (myoD), and neuronal (neurogenin1) commitment, while downregulating the self-renewal/pluripotency-associated genes Sox2, Oct4, and Nanog. REAC exposure enhanced the expression of cardiac, skeletal, and neuronal lineage-restricted marker proteins. The results obtained with murine embryonic stem cells were replicated in human adipose-derived stem cells (hADSCs) and fibroblasts. These studies revealed the feasibility of enforcing a long-lived and inexpensive commitment of hADSCs and fibroblasts to multiple complex lineages without the use of intricate chemistry or viral transduction. This opens a real perspective for the immediate and safe use of REAC in human beings. On this basis, REAC technology promises to become a new therapeutic opportunity in the field of neurodegenerative diseases that can induce both neuromodulation and regenerative processes.

### REAC efficacy in anti-senescence

Since AD is related to aging, we investigated whether REAC technology could counteract the aging process itself. Beta-galactosidase is a marker of senescence and is also related to AD. We, therefore, investigated whether REAC stimulation is effective in counteracting the expression of beta-galactosidase and of senescence-associated gene expression patterning engaged during prolonged culture of hADSCs. We demonstrated that REAC treatment remarkably influences cell viability and induces a significant downregulation of beta-galactosidase staining and the expression of senescence mediator genes. Moreover, REAC-exposed hADSCs maintained their typical fibroblast-like morphology and exhibited a multilineage potential even at late passages, as shown by the remarkable preservation of commitment to osteogenic, adipogenic, chondrogenic, and vasculogenic fates, both at the morphological and gene expression levels. We demonstrated that REAC is able to counteract degenerative senescence processes *in vitro*, as well as the biochemical and morphological changes occurring in stem cells during aging. These results support the future clinical application of REAC technology as a potential tool for blunting age-related degenerative diseases.

## Conflict of Interest Statement

Salvatore Rinaldi and Vania Fontani are the inventors of the Radio Electric Asymmetric Conveyer technology. The other authors report no conflicts of interest.

## References

[1] Manenti R Cotelli M Robertson IH Miniussi C. Transcranial brain stimulation studies of episodic memory in young adults, elderly adults and individuals with memory dysfunction: a review. Brain Stimul (2012) 5(2):103–9. 10.1016/j.brs.2012.03.004 22503472

[2] Arendash GW. Transcranial electromagnetic treatment against Alzheimer’s disease: why it has the potential to trump Alzheimer’s disease drug development. J Alzheimers Dis (2012) 32(2):243–66. 10.3233/JAD-2012-120943 22810103

[3] Boggio PS Valasek CA Campanha C Giglio AC Baptista NI Lapenta OM Non-invasive brain stimulation to assess and modulate neuroplasticity in Alzheimer’s disease. Neuropsychol Rehabil (2011) 21(5):703–16. 10.1080/09602011.2011.617943 21942868

[4] Rinaldi S Fontani V, Inventor; Rinaldi S Fontani V, assignee. Radioelectric Asymmetric Conveyer for Therapeutic Use. Patent EP1301241 (B1) (2000).

[5] Rinaldi S Fontani V, Inventor; Rinaldi S Fontani V, assignee. Radioelectric Asymmetric Conveyer for Therapeutic Use. USA Patent 7,333,859 (2001).

[6] Collodel G Moretti E Fontani V Rinaldi S Aravagli L Sarago G Effect of emotional stress on sperm quality. Indian J Med Res (2008) 128(3):254–61. 19052335

[7] Mannu P Rinaldi S Fontani V Castagna A Margotti ML. Radio electric treatment vs. es-citalopram in the treatment of panic disorders associated with major depression: an open-label, naturalistic study. Acupunct Electrother Res (2009) 34(3–4):135–49. 10.3727/036012909803861040 20344882

[8] Rinaldi S Fontani V Aravagli L Mannu P. Psychometric evaluation of a radio electric auricular treatment for stress related disorders: a double-blinded, placebo-controlled controlled pilot study. Health Qual Life Outcomes (2010) 8(1):31. 10.1186/1477-7525-8-31 20302662 PMC2850330

[9] Rinaldi S Fontani V Aravagli L Lotti Margotti M Psychological and symptomatic stress-related disorders with radio-electric treatment: psychometric evaluation. Stress Health (2010) 26(5):350–8 10.1002/smi.1298

[10] Fontani V Mannu P Castagna A Rinaldi S. Social anxiety disorder: radio electric asymmetric conveyor brain stimulation versus sertraline. Patient Prefer Adherence (2011) 5:581–6. 10.2147/PPA.S27409 22163157 PMC3234900

[11] Castagna A Fontani V Rinaldi S Mannu P. Radio electric tissue optimization in the treatment of surgical wounds. Clin Cosmet Investig Dermatol (2011) 4:133–7. 10.2147/CCID.S24090 21931498 PMC3173014

[12] Mannu P Rinaldi S Fontani V Castagna A. Long-term treatment of bipolar disorder with a radioelectric asymmetric conveyor. Neuropsychiatr Dis Treat (2011) 7:373–9. 10.2147/NDT.S22007 21822388 PMC3148928

[13] Mannu P Rinaldi S Fontani V Castagna A Margotti ML. Noninvasive brain stimulation by radioelectric asymmetric conveyor in the treatment of agoraphobia: open-label, naturalistic study. Patient Prefer Adherence (2011) 5:575–80. 10.2147/PPA.S26594 22163156 PMC3234899

[14] Olivieri EB Vecchiato C Ignaccolo N Mannu P Castagna A Aravagli L Radioelectric brain stimulation in the treatment of generalized anxiety disorder with comorbid major depression in a psychiatric hospital: a pilot study. Neuropsychiatr Dis Treat (2011) 7:449–55. 10.2147/NDT.S23420 21857785 PMC3157488

[15] Fontani V Aravagli L Margotti ML Castagna A Mannu P Rinaldi S. Neuropsychophysical optimization by REAC technology in the treatment of: sense of stress and confusion. Psychometric evaluation in a randomized, single blind, sham-controlled naturalistic study. Patient Prefer Adherence (2012) 6:195–9. 10.2147/PPA.S29734 22536055 PMC3333817

[16] Castagna A Rinaldi S Fontani V Mannu P Margotti ML. Comparison of two treatments for coxarthrosis: local hyperthermia versus radio electric asymmetrical brain stimulation. Clin Interv Aging (2011) 6:201–6. 10.2147/CIA.S23130 21822376 PMC3147051

[17] Olazaran J Gonzalez B Lopez-Alvarez J Castagna A Osa-Ruiz E Herrero-Cano V Motor effects of REAC in advanced Alzheimer’s disease: results from a pilot trial. J Alzheimers Dis (2013) 36(2):297–302. 10.3233/JAD-130077 23603397

[18] Olazaran J Gonzalez B Osa-Ruiz E Felipe-Ruiz S Boyano I Fontani V Motor effects of radio electric asymmetric conveyer in Alzheimer’s disease: results from a cross-over trial. J Alzheimers Dis (2014) 42(1):325–32. 10.3233/JAD-140417 24898637

[19] Maioli M Rinaldi S Santaniello S Castagna A Pigliaru G Delitala A Radioelectric asymmetric conveyed fields and human adipose-derived stem cells obtained with a nonenzymatic method and device: a novel approach to multipotency. Cell Transplant (2014) 23(12):1489–500. 10.3727/096368913X672037 24044359

[20] Maioli M Rinaldi S Santaniello S Castagna A Pigliaru G Gualini S Radio electric conveyed fields directly reprogram human dermal skin fibroblasts toward cardiac, neuronal, and skeletal muscle-like lineages. Cell Transplant (2013) 22(7):1227–35. 10.3727/096368912X657297 23057961

[21] Maioli M Rinaldi S Santaniello S Castagna A Pigliaru G Gualini S Radiofrequency energy loop primes cardiac, neuronal, and skeletal muscle differentiation in mouse embryonic stem cells: a new tool for improving tissue regeneration. Cell Transplant (2012) 21(6):1225–33. 10.3727/096368911X600966 21975035

[22] Rinaldi S Maioli M Santaniello S Castagna A Pigliaru G Gualini S Regenerative treatment using a radioelectric asymmetric conveyor as a novel tool in antiaging medicine: an in vitro beta-galactosidase study. Clin Interv Aging (2012) 7:191–4. 10.2147/CIA.S33312 22807628 PMC3396051

[23] Maioli M Rinaldi S Santaniello S Castagna A Pigliaru G Delitala A Anti-senescence efficacy of radio-electric asymmetric conveyer technology. Age (Dordr) (2013) 36(1):9–20. 10.1007/s11357-013-9537-8 23653328 PMC3889885

[24] Hansen N Brain stimulation for combating Alzheimer’s disease. Front Neurol (2014) 5:80 10.3389/fneur.2014.00080 24917846 PMC4040449

[25] Mura M Castagna A Fontani V Rinaldi S. Preliminary pilot fMRI study of neuropostural optimization with a noninvasive asymmetric radioelectric brain stimulation protocol in functional dysmetria. Neuropsychiatr Dis Treat (2012) 8:149–54. 10.2147/NDT.S29971 22536071 PMC3333783

[26] Rinaldi S Fontani V Castagna A. Brain activity modification produced by a single radioelectric asymmetric brain stimulation pulse: a new tool for neuropsychiatric treatments. Preliminary fMRI study. Neuropsychiatr Dis Treat (2011) 7:649–54. 10.2147/NDT.S26123 22090800 PMC3215521

[27] Rinaldi S Mura M Castagna A Fontani V. Long-lasting changes in brain activation induced by a single REAC technology pulse in Wi-Fi bands. Randomized double-blind fMRI qualitative study. Sci Rep (2014) 4:5668. 10.1038/srep05668 25011544 PMC4092330

[28] Fontani V Rinaldi S Castagna A Margotti ML. Noninvasive radioelectric asymmetric conveyor brain stimulation treatment improves balance in individuals over 65 suffering from neurological diseases: pilot study. Ther Clin Risk Manag (2012) 8:73–8. 10.2147/TCRM.S28812 22368448 PMC3284218

[29] Hsiao K Chapman P Nilsen S Eckman C Harigaya Y Younkin S Correlative memory deficits, Abeta elevation, and amyloid plaques in transgenic mice. Science (1996) 274(5284):99–102 10.1126/science.274.5284.99 8810256

[30] Calza L Baldassarro VA Giuliani A Lorenzini L Fernandez M Mangano C From the multifactorial nature of Alzheimer’s disease to multitarget therapy: the contribution of the translational approach. Curr Top Med Chem (2013) 13(15):1843–52. 10.2174/15680266113139990140 23931439

[31] Baldassarro VA Calzà L Fernández M Giardino L Giuliani A Lorenzini L Alzheimer’s disease: discussing the “bench-to-bed” and the “bed-to-bench” pathway linking preclinical and clinical research. Lost Transl (2014). p. 409–41.

[32] Balducci C Tonini R Zianni E Nazzaro C Fiordaliso F Salio M Cognitive deficits associated with alteration of synaptic metaplasticity precede plaque deposition in AbetaPP23 transgenic mice. J Alzheimers Dis (2010) 21(4):1367–81. 21504138 10.3233/jad-2010-100675

[33] Beggiato S Giuliani A Sivilia S Lorenzini L Antonelli T Imbimbo BP CHF5074 and LY450139 sub-acute treatments differently affect cortical extracellular glutamate levels in pre-plaque Tg2576 mice. Neuroscience (2014) 266:13–22. 10.1016/j.neuroscience.2014.01.065 24530449

[34] Sivilia S Lorenzini L Giuliani A Gusciglio M Fernandez M Baldassarro VA Multi-target action of the novel anti-Alzheimer compound CHF5074: in vivo study of long term treatment in Tg2576 mice. BMC Neurosci (2013) 14:44. 10.1186/1471-2202-14-44 23560952 PMC3626610

